# Triplanar U-Net with lesion-wise voting for the segmentation of new lesions on longitudinal MRI studies

**DOI:** 10.3389/fnins.2022.964250

**Published:** 2022-08-12

**Authors:** Sebastian Hitziger, Wen Xin Ling, Thomas Fritz, Tiziano D'Albis, Andreas Lemke, Joana Grilo

**Affiliations:** Mediaire GmbH, Berlin, Germany

**Keywords:** multiple sclerosis, lesion detection, longitudinal lesion segmentation, biomedical image segmentation, deep learning, MRI

## Abstract

We present a deep learning method for the segmentation of new lesions in longitudinal FLAIR MRI sequences acquired at two different time points. In our approach, the 3D volumes are processed slice-wise across the coronal, axial, and sagittal planes and the predictions from the three orientations are merged using an optimized voting strategy. Our method achieved best F1 score (0.541) among all participating methods in the MICCAI 2021 challenge *Multiple sclerosis new lesions segmentation* (MSSEG-2). Moreover, we show that our method is on par with the challenge's expert neuroradiologists: on an unbiased ground truth, our method achieves results comparable to those of the four experts in terms of detection (F1 score) and segmentation accuracy (Dice score).

## 1. Introduction

Multiple Sclerosis (MS) is a chronic, autoimmune disease which causes lesions in the central nervous system (CNS) (Kuhlmann et al., 2017). Magnetic resonance (MR) imagery is routinely used for diagnosis (Thompson et al., 2018) and prognosis (Brownlee et al., 2019) of MS by assessing the dissemination of CNS lesions in space and time. The lesions appear as white matter hyperintensities on T2 or fluid attenuated inversion recovery (FLAIR) weighted MR sequences. Tracking changes in the lesion load over time facilitates monitoring of MS activity and measuring the efficacy of disease modifying therapies (Sormani et al., 2016).

However, manually detecting and delineating lesions on MR images is a time-consuming and error-prone process with high intra- and inter-expert variability (Altay et al., 2013; Egger et al., 2017), especially when the MR acquisitions differ in terms of scanners, sequences, resolution, and quality. For these reasons, a great number of automated methods for lesion detection have been proposed and originally relied on explicit statistical features such as voxel intensities (Van Leemput et al., 2001; Lao et al., 2008; Shiee et al., 2010; Mortazavi et al., 2012; Schmidt et al., 2012; Garćıa-Lorenzo et al., 2013). However, most methods target cross-sectional segmentation and although the ISBI 2015 challenge provided longitudinal datasets, methods were not assessed on their ability to segment new or enlarging lesions (Carass et al., 2017). Existing approaches for new lesions segmentation mostly use classical image processing techniques such as image subtraction (Battaglini et al., 2014; Ganiler et al., 2014; Fartaria et al., 2019), deformation fields (Bosc et al., 2003; Salem et al., 2018), or statistical features from the independently segmented time points (Schmidt et al., 2019).

In the recent past, a number of unsupervised (Baur et al., 2021) and supervised deep learning (Zhang and Oguz, 2020; Ma et al., 2022) methods have been suggested for lesion segmentation. Especially convolutional neural networks (CNN) with encoder-decoder architectures and skip connections such as the U-Net (Ronneberger et al., 2015) have shown good performance in the ISBI 2015 and MICCAI 2016 lesion segmentation challenges (Carass et al., 2017; Commowick et al., 2018). Despite the potential of CNNs for lesion segmentation accuracy, their performance has remained below that of human experts (Carass et al., 2017; Commowick et al., 2018). In addition, deep learning based methods have only recently been designed explicitly for the segmentation of new lesions, which only appear in the follow-up but not the baseline scan. The authors of McKinley et al. (2020) independently segment both time point volumes and use the masks and confidence maps to identify new and enlarging lesions. Fully convolutional networks, in contrast, directly take as input the different time points (Krüger et al., 2020). To incorporate correlations between the different time points in the network architecture, Gessert et al. (2020b) use attention-guided interactions and (Gessert et al., 2020a) convolutional gated recurrent units. The authors of Salem et al. (2020) suggest a combined registration and new lesion segmentation network.

To foster the development of methods for assessing temporal lesion activity, the objective of the MICCAI 2021 *Multiple sclerosis new lesions segmentation* (MSSEG-2) challenge was the design of a method for automatic segmentation of new MS lesions on FLAIR MR sequences. Based on two FLAIR time points of a patient, methods had to delineate lesions that had formed on the follow-up but not on the baseline scan. The performance of the submitted algorithms was evaluated in terms of (a) their ability to detect new lesions, measured by the F1 score, and (b) the segmentation accuracy of the new lesions, measured by the Dice score. Pairs of FLAIR volumes from 40 patients were given to the challenge participants for training the algorithms, another 60 patients were held out for validation.

Our approach to this challenge starts with the observation that plain end-to-end CNNs with U-Net like architecture perform exceptionally well in most biomedical image segmentation tasks. This was clearly shown by the authors of the nnU-Net (Isensee et al., 2021), a framework which relies on either 2D or 3D U-Nets (Çiçek et al., 2016) and adjusts its hyperparameters to the given segmentation task. It achieved excellent results in many segmentation challenges, including the ISBI 2015 longitudinal lesion segmentation. While the authors found their 3D version to outperform the 2D counterpart, the performance of 2D models can be enhanced by integrating more 3D information. The triplanar or 2.5D approach processes slices across all three orthogonal directions and then merges the predictions from the different orientations (Roy et al., 2019; Henschel et al., 2020; Sundaresan et al., 2021). A triplanar approach was also used by the winner of the MICCAI 2016 challenge (McKinley et al., 2016).

In this approach, we adapt the triplanar segmentation approach and use a single 2D U-Net (Ronneberger et al., 2015) as base model. This model is trained on slices from the axial, coronal, and sagittal planes. To incorporate information from both time point volumes, corresponding slices from the two volumes are paired and given as a two-channel input. Compared to other triplanar U-Net approaches, our architecture contains two main differences:

- It uses a single U-Net which is trained on sagittal, coronal, and axial slices, allowing to share common features across orientations. This is opposed to the training of three orientation-specific U-Nets in previous approaches (McKinley et al., 2016; Roy et al., 2019; Sundaresan et al., 2021).
- For merging the predictions from different orientations, we observed that single orientation predictions tend to contain many false positive lesions. Hence, we challenge the commonly used softmax averaging and compare it to voting strategies of different sensitivity.

We submitted two segmentation pipelines to the challenge, *mediaire-A* and *mediaire-B*, which use the same model architecture but make use of different data: while the model in mediaire-A is trained only on the official training data, we use additional datasets for training the model in mediaire-B, as described below. Besides this difference, the two pipelines are identical.

Both segmentation pipelines were evaluated by the challenge organizers on the unseen test set, resulting in mediaire-B ranking 1st and mediaire-A 3rd across all submitted models in terms of detection performance (F1 score). In additional validations, where we compare our pipelines to the challenge's annotators, we show that our algorithms are on par with the neuroradiologists in terms of F1 score and segmentation accuracy (Dice score).

## 2. Materials and equipment

The majority of the 3D FLAIR images used in this study for training and testing the models was provided by the MSSEG-2 challenge organizers. In addition, 25 internal datasets with pairs of 3D FLAIR images were used for training and validation. All used data, including the corresponding ground truth masks, is described in the following paragraphs. An overview of the datasets is provided in Table 1.

**Table 1:** Datasets used for training, validation, and testing, provided by the MSSEG-2 challenge organizers and internal data.

**Name**	**Source**	**No. of patients**	**Sequence**	**Voxel resolutions**
TRAIN-MSSEG2	OFSEP HD	40 (29)	3D FLAIR	0.5–1.2 mm anisotropic
TEST-MSSEG2-NL	OFSEP HD	32	3D FLAIR	0.5–1.2 mm anisotropic
TRAIN-B-NL	Internal	25	3D FLAIR	0.5–1.2 mm anisotropic
VAL-A-NL	Internal	20	3D FLAIR	0.5–1.2 mm anisotropic

The suffix -NL denotes that all datasets exhibit new lesions, otherwise the number of patients with new lesions is denoted in parentheses. Each dataset contains 3D FLAIR images of a patient, a baseline and a follow-up scan. Note that the internal datasets in VAL-A-NL are a subset of TRAIN-B-NL. The datasets are described in more detail in Section 2.

### 2.1. MSSEG-2 datasets

The data provided by the organizers of the MSSEG-2 challenge consists of 100 pairs of 3D FLAIR weighted MRI sequences from the OFSEP HD cohort^1^, each corresponding to two scans of the same patient acquired at different time points (1–3 years apart). The images had been acquired on 15 MRI scanners from different manufacturers (GE, Philips, Siemens) in different locations and exhibited varying resolutions and anisotropic voxel sizes, with resolutions between 0.5 and 1.2 mm. Besides the 3D FLAIR sequences, no other sequences were used for the creation of the ground truth or provided to the participants. For each data pair, a consensus ground truth mask was created from the delineations of four expert neuroradiologists using the protocol described in the following paragraph. Forty of the 100 3D FLAIR image pairs were provided to the challenge participants for training their models, together with the four experts' new lesion segmentations and the consensus ground truth masks. We will refer to these datasets as *TRAIN-MSSEG2*. The remaining 60 pairs were used for evaluating the submitted models. These datasets, including consensus ground truth and the experts' segmentation masks, were provided to the participants after publication of the official challenge results for further analysis. For the calculation of the challenge's main metrics, i.e., the Dice and the F1 score, only the 32 of the 60 dataset pairs that exhibited new lesions were taken into account. We will denote this subset, which is used for the evaluations in Section 4, as *TEST-MSSEG2-NL*. The remainder of the MSSEG-2 test datasets were used by the challenge organizers for further evaluations which are outside the scope of this study and are not used here. The information on data, data access, and annotations is also available on the challenge websites.^2–4^

#### 2.1.1. Consensus reading protocol

For every dataset, manual delineations of new lesions were performed by four expert neuroradiologists, medically trained for MS and at the start of their career (a few years after taking their permanent position). They received instructions to delineate lesions not in contact with other lesions and above 3 mm in size in one of the image planes. The delineation was performed using the software ITK Snap, for which the experts had received a user manual.

Based on the resulting four expert segmentation masks, a consensus ground truth was created with the help of a senior expert neuroradiologist with much longer experience in neuroradiology and MS than the other four experts. The ground truth creation was done in two steps: (i) lesion approval or rejection and (ii) delineation. In step (i), every *majority lesion*, i.e., found by at least three of the four experts, automatically transferred to the ground truth; for any *disputed lesion*, i.e., found by at most two of the experts, the senior expert decided whether to accept or reject it. In step (ii), the delineation of every *accepted lesion* was calculated using the STAPLE (Akhondi-Asl and Warfield, 2013) algorithm based on the concerned experts' lesion segmentations.

As the ground consensus ground truth masks were created by the experts, a direct evaluation of the experts on this same ground truth would be biased. Therefore, we additionally created expert-specific unbiased ground truth masks to compare our pipelines to the experts (see Section 3.6).

### 2.2. Internal datasets

While our model in pipeline *mediaire-A* was trained only on the challenge's official 40 patient volumes, we added internal datasets to train pipeline *mediaire-B*. These consisted of 25 pairs of all 3D FLAIR images from 25 patients, where each pair exhibited new lesions, and will be referred to as *TRAIN-B-NL*. The datasets had been acquired on different scanners by Siemens (Aera 1.5 T, Magnetom Vida 3.0 T, Skyra 1.5 T, Skyra 3.0 T) and Philips (Achieva 1.5 T, Achieva 3.0 T) and had anisotropic voxel resolutions between 0.5 and 1.2 mm. In order to match the challenge data, we also only used the 3D FLAIR sequences for ground truth creation and model training without any additional sequences. Each of the image pairs was annotated by up to four experts (a medical doctor and neuroscientist, a radiologist, and two radiographers with special training in segmenting MS lesions, all of them with more than 2 years of experience with MS-specific MRI interpretation and annotation), and a consensus ground truth had been formed similar to the one used in the challenge, as described in Section 2.1.1. The segmentations were performed using an annotation application integrated into an internal image viewer.

As we trained the models in pipelines mediaire-A and mediaire-B on 5 data folds (see Section 3.1.2) with random 80–20% train-validation splits, we validated the individual fold models on these validation splits. However, for the validation of the orientation merging strategies (cf. Section 3.4), we required the final ensemble model of all folds. For this purpose, we used a subset of 20 patients from TRAIN-B-NL. Since pipeline mediaire-B was trained on these datasets, they could only be used to validate mediaire-A and we denote them as *VAL-A-NL*. We assume that the results of comparing the orientation merging strategies transfer qualitatively from mediaire-A to mediaire-B, as the pipelines are very similar.

### 2.3. Pre-processing

For each patient in the datasets provided by the MSSEG-2 challenge, the organizers had transformed the two scans onto a common middle point through rigid registration.

We further applied the following preprocessing steps to all 3D FLAIR image pairs in the challenge's and internal training, validation, and test sets: (1) affine registration of each pair of 3D FLAIR images to the MNI template, (2) cropping the FOV to an area around the brain, (3) resampling the volume to 256 × 256 × 256 voxels, and (4) pixel normalization through mean subtraction and division by the standard deviation.

To increase the generalization ability of the model, data augmentation was performed on the preprocessed 3D volumes of the training sets during training, including contrast augmentation, rotations, flipping across the three orthogonal planes, elastic deformations, and bias field augmentation.

## 3. Methods

**Figure 1:**
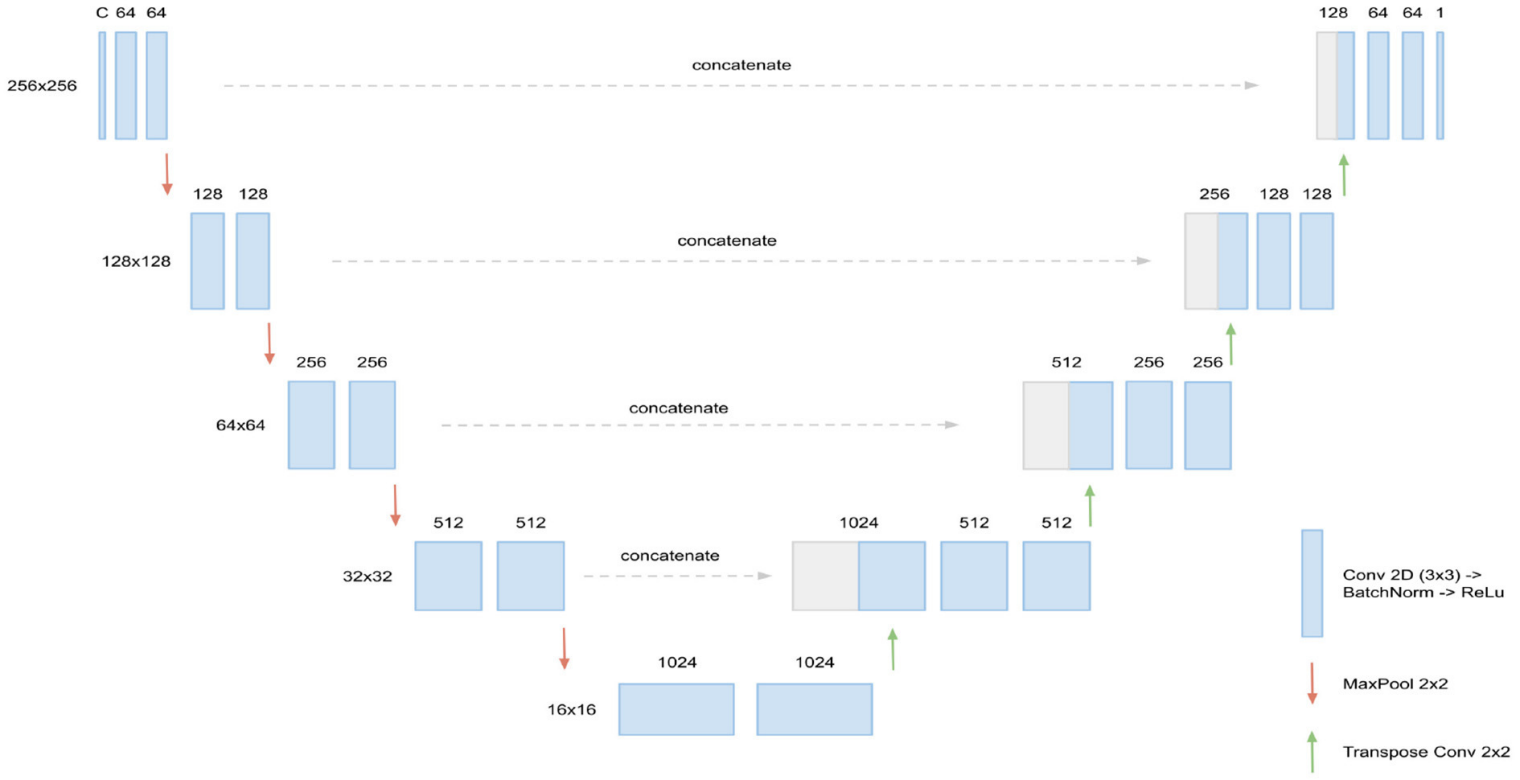
Architecture of the 2D U-Net used. Input are *C* = 2 slices (axial, coronal, or sagittal) corresponding of dimension 256 × 256, corresponding to the two FLAIR time points. In every layer of the encoding branch (left), two convolution blocks, consisting of Conv2D (3 × 3), BatchNorm, and ReLU activation, are applied. When passing to a new layer, dimensions are reduced to half by max pooling while the number of channels is doubled in the first convolution block. In the decoding branch (right), the max pooling operations are replaced by transpose convolutions for upsampling and the data from the corresponding layer of the encoding branch is concatenated through skip connections. Output of the U-Net is the 256 × 256 binary mask containing the new lesions segmentation.

The basis for our segmentation pipeline, which we refer to as triplanar U-Net, is a 2D U-Net (Ronneberger et al., 2015). It has two input channels with corresponding slices—either axial, coronal, or sagittal—from the two different time points of each patient. The output of the model is a single-channel 2D binary mask, representing the segmentation of the new lesions found in the corresponding slice. For an illustration and the dimensions of the network (see Figure 1).

Compared to previously suggested triplanar U-Net architectures (Roy et al., 2019; Sundaresan et al., 2021), our approach has two main differences:

1. It uses a single U-Net which is trained on sagittal, coronal, and axial slices, allowing to share common features across orientations. This is opposed to training three orientation-specific U-Nets in the former approaches. Note that this procedure requires the all slices to be of the same dimensions, which is ensured by resampling the volume to a regular cube, as described in Section 2.3.
2. For merging the predictions from different orientations, we test different techniques. In addition to softmax averaging (i.e., averaging the predicted probabilities), we implement and validate three voting strategies of different sensitivities to optimize the method's recall and precision. The best strategy is then implemented.

### 3.1. Model training

The U-Nets trained for pipelines mediaire-A and mediaire-B use exactly the same training protocol and hyperparameters. However, only the 40 datasets in TRAIN-MSSEG2 were used for training mediaire-A, while mediaire-B was trained on TRAIN-MSSEG2 plus the additional 25 datasets in TRAIN-B-NL (see Section 2).

We trained the triplanar U-Net on batches, each combining a total of 20 axial, coronal, and sagittal slices from different patient volumes for robustness. For the updates of the model weights, we used stochastic gradient descent with momentum and an initial learning rate of 0.0001, which was reduced when the validation loss plateaued. Training was performed with early stopping when the validation loss stopped decreasing, which was usually the case after around 50 epochs.

#### 3.1.1. Loss function

Recently, it has been observed that combined loss functions tend to be more robust and accurate, especially in segmentation tasks with high class imbalance. For instance, the self-configuring segmentation network nnU-Net (Isensee et al., 2021) uses the combo loss as a default, which is the sum of the Dice loss and the cross entropy loss. For training our models, we use a combination of Dice loss and the TopK loss (Wu et al., 2016), which has shown good performance, for example in the winning and runner up model (Ma, 2021) of the Miccai 2020 ADAM segmentation^5^ challenge. The TopK is a hard-mining variant of the cross-entropy loss, focussing only on the *k*% hardest voxels. We denote with *g*_*ic*_∈{0, 1}, *p*_*ic*_∈(0, 1) the ground truth index and the softmax prediction for voxel *i* and class *c*, respectively, and by select_top_*k*_ the function that returns the *k*% largest values. Then the partial loss functions *L*_Dice_, *L*_TopK_, and the total loss function *L*_total_ are defined by


$$\begin{array}{l} L_{\text{Dice}}=1-\frac{2\sum_{i,c}g_{ic}\cdot p_{ic}}{\sum_{i,c}g_{ic}^{2}+\sum_{i,c}p_{ic}^{2}}\\ L_{\text{TopK}}=-mean(select\_top_{k}(S_{CE}))\\ L_{\text{total}}=L_{\text{Dice}}+L_{\text{TopK}} \end{array}$$

where *S*_*CE*_ = {_*g*_*ic*_log(*p*_*ic*_)}*i, c*_ is the set of cross entropy scores for all voxels and classes. Note that *L*_TopK_ reduces to the cross entropy loss for *k* = 100. In our experiments, we chose *k* = 10.

#### 3.1.2. Cross validation

For each pipeline, mediaire-A and mediaire-B, we train the triplanar U-Net five different data folds, resulting in models *M*_0_, ..., *M*_4_. For each *M*_*i*_, we hold out 20% of the training data. For inference, the ensemble of all five fold models will be used for segmentation, as explained in Section 3.2.

### 3.2. Inference

**Figure 2:**
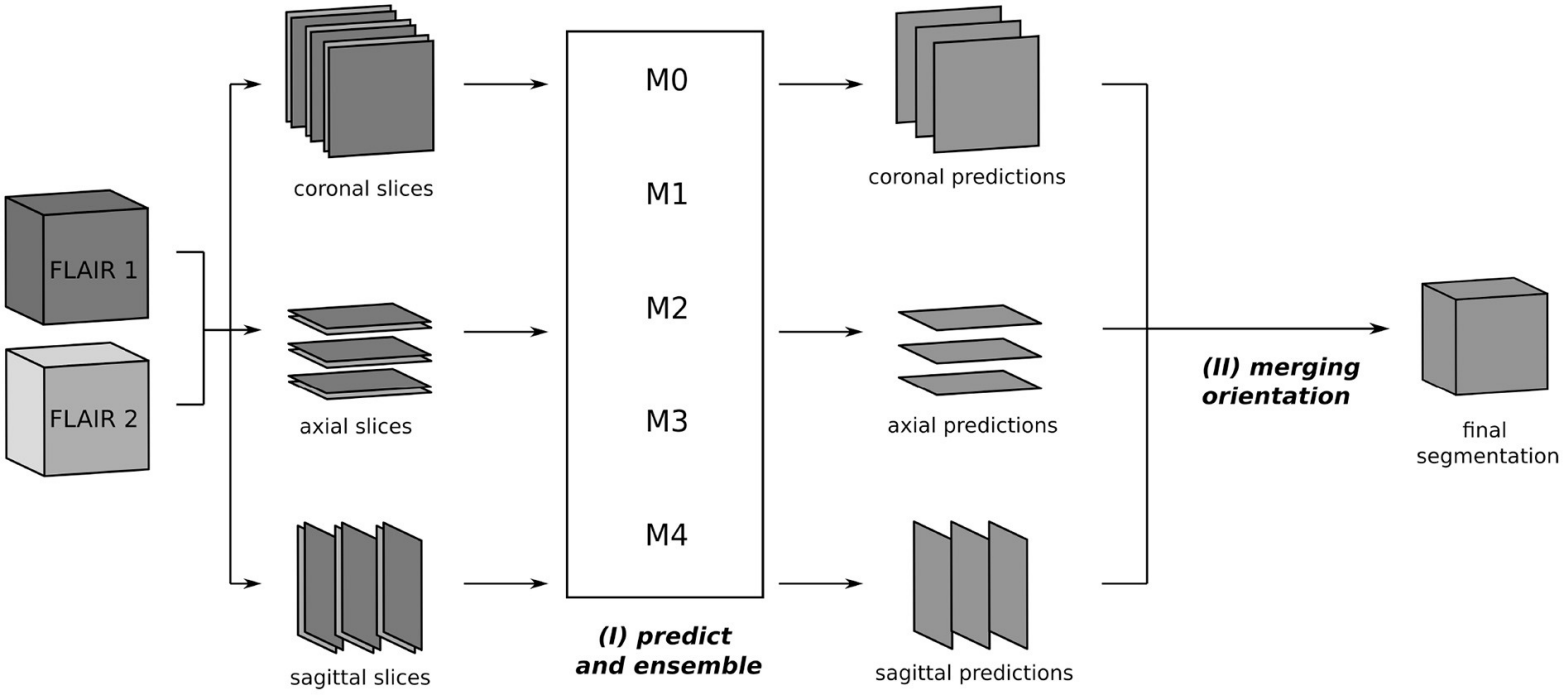
Segmentation process using the triplanar U-Nets *M*_0_, ..., *M*_4_ trained on slices from the three orthogonal planes of the different training folds. The 3D FLAIR input volumes are sliced along the coronal, axial, and sagittal planes and grouped together in pairs of corresponding slices. For every orientations, the segmentation is now performed independently: (I) each slice pair is given as a two-channel 2D input to the models *M*_0_, ..., *M*_4_ and predicted softmax scores are averaged. (II) In the final step, the predictions of the individual orientations are merged to yield the final segmentation.

The segmentation process at inference is depicted in Figure 2. From the two 3D FLAIR volumes of each patient, three datasets are created, consisting of pairs of axial, coronal, and sagittal slices, respectively. For each such dataset, inference is performed slice-wise with every fold model *M*_0_, ..., *M*_4_ and the ensemble average of the resulting softmax slices is calculated, resulting in axial, coronal, and sagittal predictions. Then, the three single-orientation predictions are merged to produce the final segmentation mask. This is explained in detail in Section 3.4, where different merging strategies are compared.

### 3.3. Metrics

To evaluate our experiments, we use the official performance metrics from the MSSEG-2 challenge. These are defined *via* the true positives (TP), false positives (FP), and false negatives (FN) on the lesion level (TP_l_, …) and the voxel level (TP_v_, …). The principal new lesion *detection metric* is the F1 score, but we also investigate precision and recall. They are defined as


$$\begin{array}{l} \;\text{F}1\;\text{score}=\frac{2*{\text{TP}}_{\text{l}}}{{\text{FP}}_{\text{l}}{+2*\text{TP}}_{\text{l}}{\text{+FN}}_{\text{l}}}\\ \;\text{Recall}=\frac{{\text{TP}}_{\text{l}}}{{\text{TP}}_{\text{l}}{\text{+FN}}_{\text{l}}}\\ \text{Precision}=\frac{{\text{TP}}_{\text{l}}}{{\text{TP}}_{\text{l}}{\text{+FP}}_{\text{l}}} \end{array}$$

The principal *segmentation metric* used is the Dice score, which is the equivalent of the F1 score on a voxel level:


$$\text{Dice}\;\text{score}=\frac{2*{\text{TP}}_{\text{v}}}{{\text{FP}}_{\text{v}}{+2*\text{TP}}_{\text{v}}{\text{+FN}}_{\text{v}}}$$

We note that the quantities TP_l_, FP_l_, andFN_l_ depend on the definition of when lesions in the prediction and the ground truth shall be matched. In the competition evaluation, a match requires certain overlap thresholds to be fulfilled. This is described in detail in the official documentation^6^. All metrics in this paper were calculated using the “animaSegPerfAnalyzer” command from the Anima toolbox^7^, which was also used by the challenge organizers to calculate the official results for the leaderboard.

### 3.4. Validation of orientation merging strategies

As described in Section 3.2, the inference pipeline requires to merge predictions from different orientations. While softmax averaging is commonly used for this step (McKinley et al., 2016; Roy et al., 2019; Sundaresan et al., 2021), we additionally compare three different voting strategies in order to find the optimal balance of recall and precision. This step is depicted in Figure 2(II).

Starting from the predicted probability maps (softmax scores) of each orientation, we first calculated the softmax average as a baseline approach. For the other approaches, which operated on a lesion level, we first thresholded the softmax scores of each of the three orientations to yield hard predictions. Then three different lesion-selection strategies were applied: A lesion was predicted if detected in (a) at least one orientation (union); (b) at least two orientations (majority); (c) all orientations (unanimous voting). The exact segmentation of each selected lesion was defined as the union of the corresponding positive voxels across orientation predictions.

The four approaches were implemented into pipeline mediaire-A and used for segmenting the internal datasets VAL-A-NL (see Section 2.2). The segmentation masks were then rated against the corresponding expert annotations and the results in terms of F1 score, precision, recall, and Dice are shown in **Figure 4**. The optimal strategy was chosen based on the best F1 score. As mediaire-B was trained on datasets containing VAL-A-NL, this validation could not be performed directly for this pipeline. However, due to the similarity of both pipelines, it was assumed that the optimal strategy for mediaire-A would also be the best strategy for mediaire-B.

### 3.5. Challenge evaluation: Comparison to other participants

Both pipelines, mediaire-A and mediaire-B were submitted to the challenge among a total of 29 rated submissions from 24 teams. For all submitted pipelines, the organizers calculated the predictions on the test dataset with 60 patients. However, for calculating the scores on the detection (F1 score) and the segmentation leaderboard (Dice score), only the 32 patients in TEST-MSSEG2-NL, i.e., those with at least one new lesion, were taken into account (cf. Section 2.1).

The official raw scores for all submissions are publically available^8^. In addition to the official average scores across patients, we visualize the score distributions across patients.

### 3.6. Challenge evaluation: Comparison on unbiased ground truth

For assessing how our pipelines mediaire-A and mediaire-B perform compared to human neuroradiologists, we evaluate the lesion delineations conducted by the four challenge experts. A naive approach would simply rate the human segmentation masks against the consensus ground truth, as it has been done for the segmentation masks produced by the algorithms. In fact, the resulting scores from this approach are published by the challenge organizers and correspond to those in **Figure 5**. However, this approach comes with a problem: The ground truth has been created based on the individual segmentation masks of the human experts, which makes it biased toward these experts. Thus, the measured human performance is likely to be higher than it would be on an unbiased ground truth.

We therefore suggest a comparison on an unbiased ground truth of the official challenge test datasets with new lesions, TEST-MSSEG2-NL (cf. Section 2.1), constructed from the corresponding experts' segmentation masks and the consensus ground truth masks, which were provided to the participants after the challenge. For a fair comparison, the segmentation mask *s*_*i*_ of Expert *i* should be rated against a ground truth *u*_*i*_ whose definition is independent of *s*_*i*_. We create *u*_*i*_ from the segmentation masks of all other experts $S_{\bar{i}}=\left\{s_{j}|j\neq i\right\}$ using the challenge's consensus reading protocol, as described in Section 2.1.1. By doing so, we exclude the minimal information necessary (segmentation *s*_*i*_) to unbias the ground truth while preserving the maximal expert knowledge available (segmentations $S_{\bar{i}}$ and senior expert decisions). The protocol involves (i) the acceptance or rejection of lesions found by any expert and (ii) calculating the segmentation of each accepted lesion through majority voting. While (ii) is a simple voxel-wise calculation, the decisions (i) on disputed lesions are taken by a senior expert. We cannot consult the senior expert, however, we can derive the decisions as they are implicitly contained in the consensus ground truth *c*. The only assumption we make for this derivation is that of constant decisions: if a disputed lesion *l* was approved (rejected) by the senior expert in the original reading, this same lesion *l* is also approved (rejected) in a different reading (where the number of total expert masks may be different).

**Figure 3:**
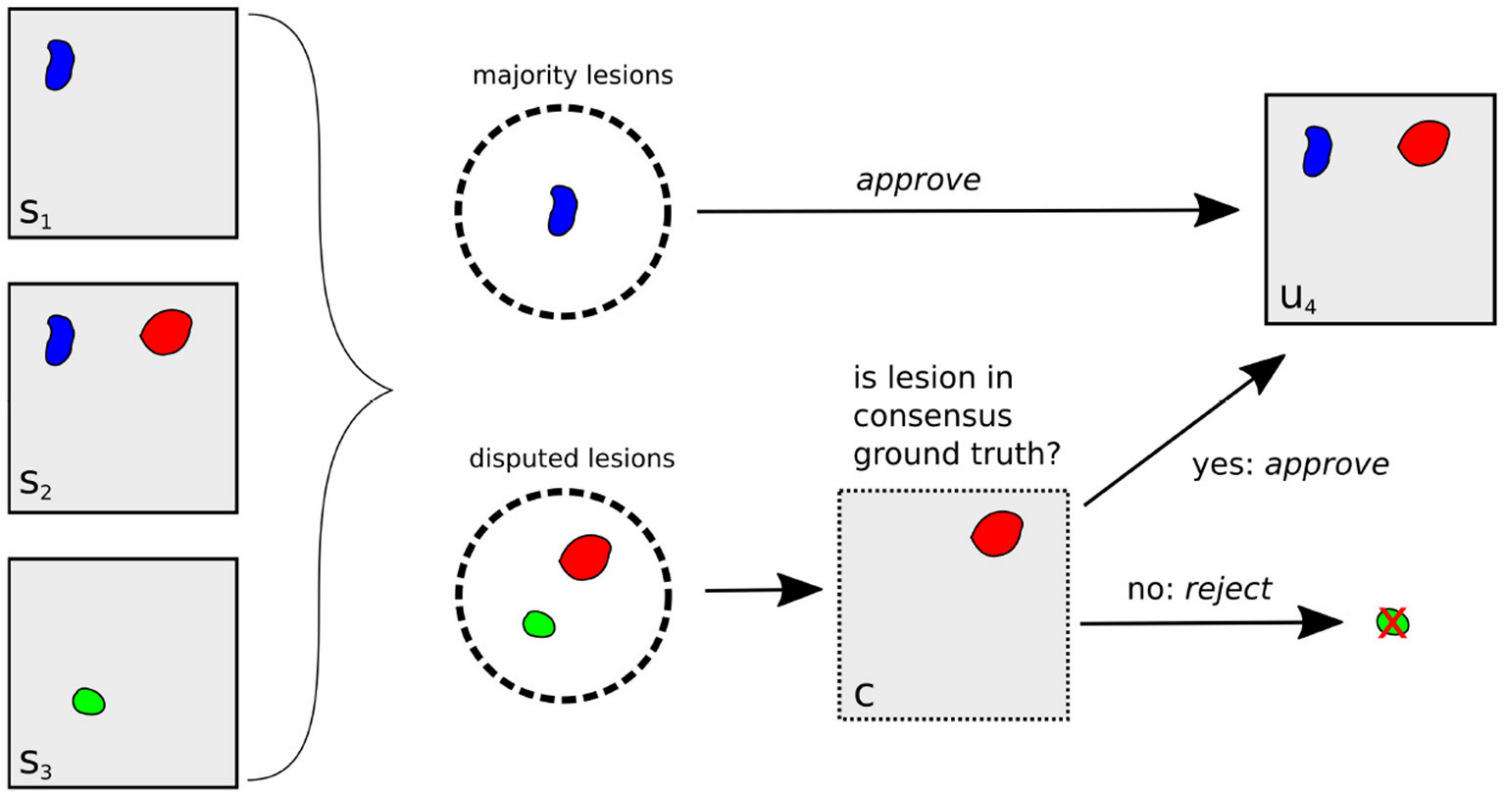
Illustration of unbiased ground truth creation *u*_4_ for Expert 4, (right) from the segmentation masks of experts 1, 2, and 3 (left). If a lesion is found by at least two experts (blue lesion), it is automatically selected for *u*_4_. Otherwise, it is a disputed lesion (green, red) and has to be decided on by the senior expert. If it is contained in the consensus ground truth *c* (red lesion), it has been accepted by the senior expert before, so we approve it. Otherwise, it is rejected.

The complete lesion selection process (i) is illustrated in Figure 3 for the example of creating an unbiased ground truth *u*_4_ for Expert 4: First, all lesions in the segmentation masks *s*_1_, *s*_2_, *s*_3_ are grouped into *majority lesions* (found by at least two experts) and *disputed lesions*. The majority lesions are automatically accepted according to the protocol (cf. Section 2.1.1). If a lesion *l* is disputed, i.e., found by a single expert, it must have been found by at most two experts in the original reading (as this reading had an additional expert). Hence, it was already a disputed lesion in the original reading (cf. Section 2.1.1) and we can derive the senior expert's decision from the consensus ground truth *c*: if *c* contains lesion *l*, it has previously been approved and we include it into the unbiased ground truth. Otherwise, it has previously been rejected and we exclude it.

Having selected all relevant lesions, their exact segmentations are calculated as (ii) the voxel-wise majority vote across the segmentation masks *s*_1_, *s*_2_, *s*_3_, resulting in the unbiased ground truth *u*_4_.

We apply the protocol (i, ii) defined above to generate unbiased ground truth masks *u*_1_, …, *u*_4_ for all experts and all patients in the test set. Each expert *i* is now evaluated by rating the segmentation *s*_*i*_ against *u*_*i*_ in terms of recall, precision, F1 score, and Dice score. As each expert is now rated against a different ground truth, their scores are not directly comparable. Hence, for every expert *i*, we also rate pipeline mediaire-A and mediaire-B on *u*_*i*_ and compare the resulting scores to this expert. From these four individual assessments, we then calculate the mean scores for experts, mediaire-A, and mediaire-B to compare the average performance model vs. human performance.

#### 3.6.1. Statistical testing

In order to assess whether our pipeline performance is comparable to or better than the expert performance, we tested for statistical significance. To this end, we first defined a margin *d* = 0.05 and regarded performances as *comparable* if their absolute difference was below *d*. If we wanted to test for comparability only, we could use equivalence tests with margin *d*. However, since we want to investigate if the models are comparable *or better* than the experts, we choose to conduct *noninferiority tests* (Walker and Nowacki, 2011) with margin *d*. This leads to the null hypothesis *H*_0_ that the expected difference *E*(*Y*−*X*) between expert performance *Y* and pipeline performance *X* is above *d*. We assess the validity of the null hypothesis *H*_0_ using paired difference Student's *t*-tests with significance level α = 0.05. The tests are conducted for F1 score and Dice and for every combination of pipeline and expert. In addition, we test the pipelines average performance across the different masks *u*_*i*_ against the average performance across experts.

### 3.7. Implementation

The model is implemented and trained in Python using the PyTorch package.

## 4. Results

### 4.1. Validation of orientation merging strategies

**Figure 4:**
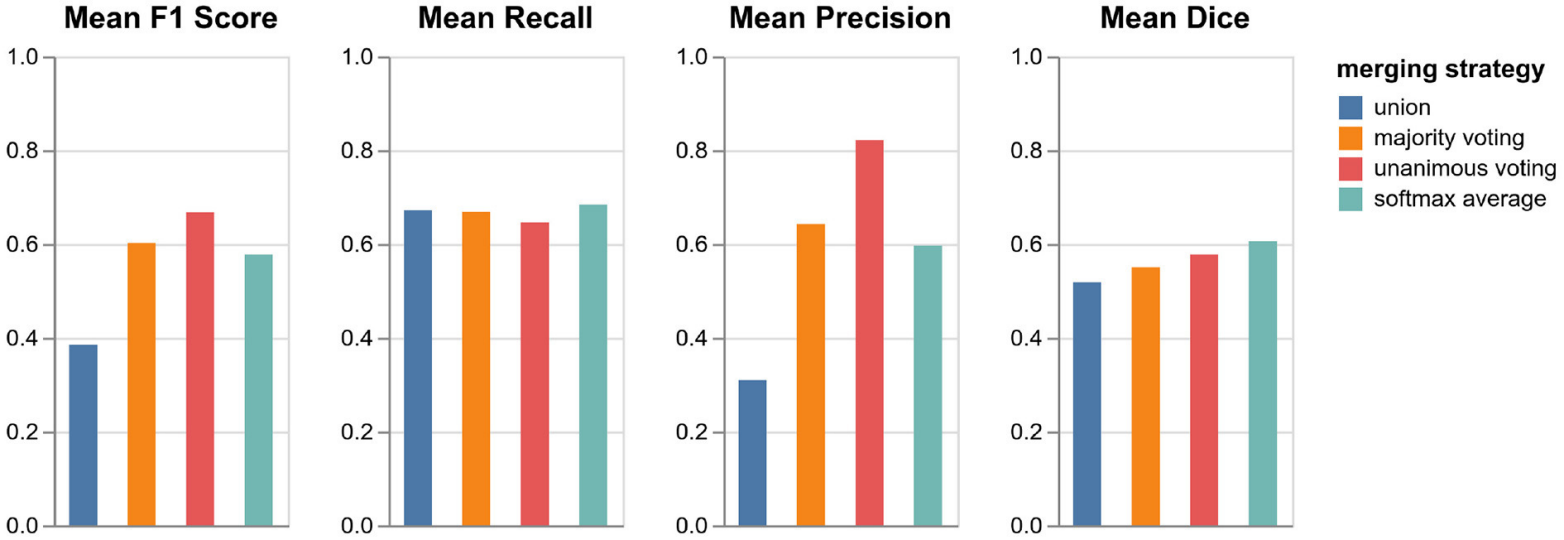
Performance of pipeline mediaire-A with different strategies for merging the predictions from axial, coronal, and sagittal orientation. Scores are calculated on the 20 datasets in VAL-A-NL. The approaches union, majority voting, unanimous voting, and softmax averaging are compared in terms of F1 score, precision, recall, and Dice.

As described in Section 3.4, we tested four different strategies for merging the predictions from the three orthogonal orientations: three lesion voting procedures and softmax averaging. Figure 4 shows the performances of the respective methods validated on the datasets in VAL-A-NL (Section 2.2). The most inclusive strategy, union of all lesions, achieves the best recall but a very low precision and thus a bad overall F1 score. While majority voting is significantly better, unanimous voting clearly achieves best F1 score due to a high precision. The baseline method, softmax averaging, shows a performance similar to majority voting. It is only in terms of segmentation accuracy (Dice), that softmax averaging outperforms all voting strategies.

It is interesting that the precision gain when using the very restrictive unanimous voting strategy largely outweighs the slight loss in recall. Apparently, the weakness of a single-orientation model is not its capability to find enough lesions—it rather bears the risk of classifying too many confounding hyperintensities as lesions. The unanimous voting strategy could also be reformulated as: Accept only lesions which have been “seen” in all three orientations.

Since the focus of the challenge is on the detection performance and the most important metric is the F1 score, we implement unanimous voting in both pipelines mediaire-A and mediaire-B.

### 4.2. Challenge evaluation: Comparison to other participants

**Figure 5:**
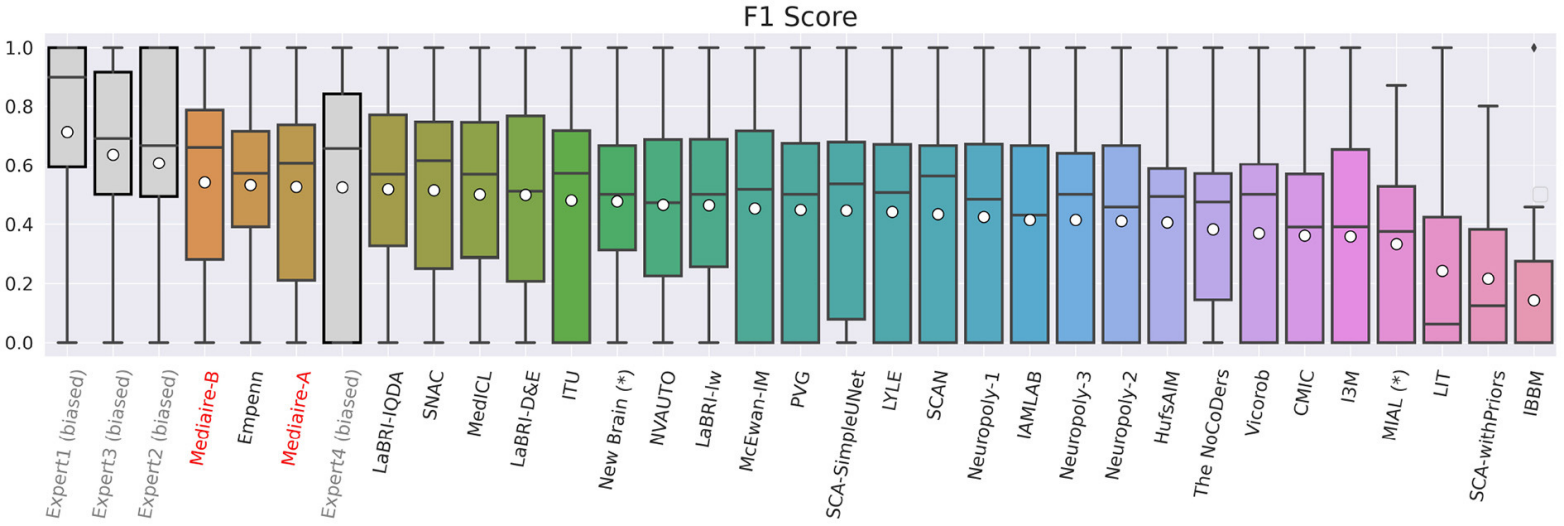
F1 scores of experts (gray), our pipelines mediaire-A and mediaire-B (red), and the models submitted by the other MSSEG-2 participants (black), calculated on the 32 datasets in TEST-MSSEG2-NL, all of which exhibited new MS lesions. Horizontal bars indicate the median and white circles the mean values. All experts and models are ordered by their mean F1 score, which also determined the ranking of the main challenge leaderboard. The three best performing methods are our pipeline mediaire-B, Empenn, and pipeline mediaire-A. The scores of the expert segmentations are shown for reference, however, these scores are biased as discussed in Section 3.6.

The boxplot in Figure 5 shows the official F1 scores of the challenge's main leaderboard for all 29 submissions rated against the consensus ground truth of the datasets in TEST-MSSEG2-NL. It also includes the scores obtained by the experts' segmentation masks when rated against the consensus ground truth. As discussed in Section 3.6, the latter scores are positively biased, as the rated segmentation masks were the basis for the ground truth creation. An unbiased comparison between our submissions and expert performance is therefore done in Section 4.3.

In terms of detection performance (F1 score), the three best methods are mediaire-B (0.541), Empenn (0.532), and mediaire-A (0.525), respectively. The second best submission Empenn performed segmentation with a 3D nnU-Net (Isensee et al., 2021) trained on official and internal datasets. The great majority of submissions, including all top 10 methods, used deep learning with 3D or 2.5D U-Net-like architectures.

### 4.3. Challenge evaluation: Comparison on unbiased ground truth

**Figure 6:**
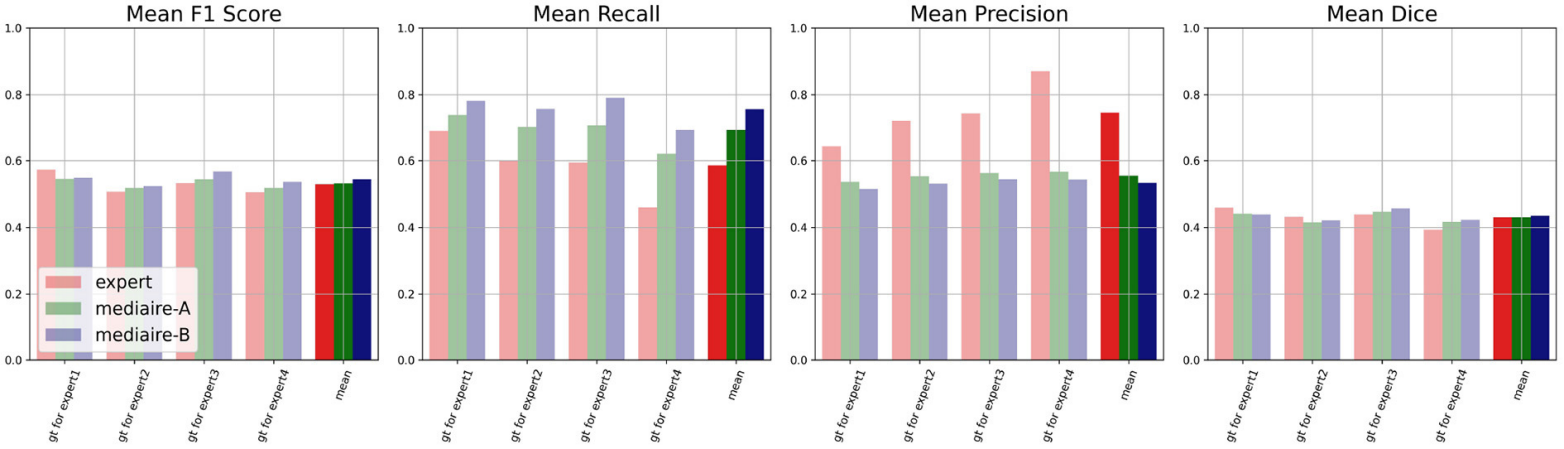
Unbiased comparison of each of the experts 1, …, 4 to segmentation pipelines mediaire-A and mediaire-B, in terms of F1 score, recall, precision, and Dice. The scores are calculated on the unbiased ground truth masks of the 32 patients in TEST-MSSEG2-NL. For every expert, this unbiased mask is constructed from the other expert masks (cf. Section 3.6) resulting in four individual comparisons with different ground truths. In the last block of each plot, we show the average score across the four experts against the average score of each pipeline across the different ground truth masks. Clearly, the segmentation pipelines have a higher recall while the experts have higher precision. In terms of F1 and Dice scores, the pipelines achieve slightly higher average results.

The results of the comparison between algorithms and experts on the unbiased ground truth of the TEST-MSSEG2-NL data (cf. Section 4.3) are shown in Figure 6. Clearly, both pipelines mediaire-A and mediaire-B have higher recall but lower precision than the experts (second and third plot, respectively). In the overall detection performance, the algorithms slightly outperform the experts on average (first plot, last block) and only Expert 1 achieves a slightly higher F1 score. This is in contrast to the evaluation on the (biased) consensus ground truth in Section 4.2, where experts 1, 2, and 3 had significantly higher F1 scores than all submitted methods. In terms of segmentation accuracy (last plot), expert and algorithm performances are very similar.

The differences in F1 score and Dice between experts and models are relatively small and statistically not significant. We therefore tested for non-inferiority, i.e., if each pipeline's performance is within a *d* = 0.05 margin or better than each expert's performance using paired t-tests, as described in Section 3.6.1. The resulting *p*-values are shown in Table 2 with the significant values (*p* < 0.05) in bold. In terms of F1 score, the results for mediare-A are significant only when compared to Expert 2, while for mediaire-B they are significant when compared to expert experts 2, 3, 4 and the average across experts. In terms of Dice score, test results for mediaire-A and mediaire-B are significant when compared to experts 2 and 4 and the mean of experts.

**Table 2:** *p*-values for testing non-inferiority of the performance of a pipeline (rows) compared to an expert (columns) with a margin of *d* = 0.05.

		**Expert 1**	**Expert 2**	**Expert 3**	**Expert 4**	**Mean**
mediaire-A	F1 score	0.3186	**0.0367**	0.1160	0.1015	0.0551
	dice	0.2037	**0.0447**	0.0873	**0.0261**	**0.0161**
mediaire-B	F1 score	0.2935	**0.0248**	**0.0361**	**0.0326**	**0.0135**
	dice	0.2254	**0.0245**	0.0532	**0.0204**	**0.0101**

Significant p-values (p < 0.05) are marked in bold. The last column shows the p-values for the scores averaged across the different experts. The values are calculated using paired t-tests using the pipeline and expert scores on the 32 datasets in TEST-MSSEG2-NL.

In conclusion, we showed that our better pipeline, mediaire-B, is at least comparable to three (two) of the four experts and the expert average in terms of F1 score (Dice score).

Processing of the segmentations took an average of 97 s per dataset (±2 s standard deviation) on a Laptop with graphics processing unit (CPU: Intel Core i7-10750H, 32 GiB RAM; GPU: NVIDIA GeForce RTX 2080 Super, 8 GiB RAM).

## 5. Discussion

The detection of new MS lesions is clinically important for diagnosis, prognosis, and treatment monitoring. An automatic method with a detection and segmentation accuracy comparable to that of an expert neuroradiologist can be highly beneficial to improve diagnostic quality by providing a “second pair of eyes,” to decrease inter-rater variability, and to reduce the manual reading time and effort. For instance, the study in Altay et al. (2013) assumed a maximal time of 10 min for a clinician to count lesions on an MS dataset and showed significant variability in the results of clinicians of different expertise level.

We presented a deep learning based approach using the U-Net to segment new lesions on 3D FLAIR volumes by processing slices from axial, coronal, and sagittal planes. We showed that our U-Net based segmentation pipelines not only outperform all other competing methods in the MSSEG-2 challenge in terms of detection accuracy measured by lesion-wise F1-score. They are also on par with an average expert neuroradiologist, both in detection (F1 score) and segmentation accuracy (Dice score) when compared on an unbiased ground truth. The automatic lesion segmentation was performed in <2 min on a Laptop with GPU, which is significantly less than the expected annotation time needed by a human annotator.

As a major difference to other triplanar or 2.5D U-Nets with softmax averaging of orientations, our algorithm uses unanimous voting which only accepts lesions that have been confirmed in all three orientations. Even though this approach may seem restrictive, it is actually aligned with the diagnostic guideline for MS lesions detection that lesions should be confirmed on multiple planes to avoid false positive results (Filippi et al., 2019). In addition, we saw in Section 4.3 that our algorithm outperformed the human experts in recall but had lower precision. For any less restrictive strategy than unanimous voting, this discrepancy would have been even more severe, which also becomes clear from the validation in Figure 4. We therefore suggest that unanimous voting is a key factor for the good performance of our algorithm.

Another slight performance gain was achieved through the use of additional training data, leading to a higher recall of the model mediaire-B compared to mediaire-A (cf. Figure 6). While the augmentation of the training size does not always lead to improved model performance in our experience, we took particular care to optimize the distribution of the additional data: (i) we added only patients with new lesions, leading to a recall improvement with only slight decrease in precision, and (ii) the corresponding consensus ground truth was created using a protocol similar to the one used by the challenge organizers.

While the presented outcomes are encouraging, there is still room for improvement: our algorithms had a higher recall than the average neuroradiologist, however, the precision was lower. Future works may therefore focus on an improved false positive reduction. Furthermore, we could observe a performance gain by increasing the relatively small training set from 40 (mediaire-A) to 65 datasets (mediaire-B). Training on a larger set could therefore increase performance even further.

Another limiting factor of this study is the use of only 3D FLAIR datasets acquired with high resolution which does not necessarily reflect the clinical reality. While the presented approach can be applied to 2D, low-resolution, or low-quality datasets, we do not know how well the present results translate to such a data regime. In particular, the information in thick slices may not be sufficient to distinguish a lesion from brain tissue. To this end, we suggest a follow-up study with a larger and more diverse training and test set in order to yield a complete assessment covering a broad range of clinical settings.

## Code availability statement

The code for training the models in pipelines mediaire-A and mediaire-B and performing the segmentations is proprietary and not publically available. For studies involving the comparison to or the reproduction of the presented results, the trained models may be provided within a research collaboration. In this case, researchers are invited to reach out to the CEO of mediaire, Andreas Lemke (a.lemke@mediaire.de).

## Data availability statement

The data analyzed in this study is subject to the following licenses/restrictions: The data for training pipeline mediaire-A and calculating the results was obtained as part of the MICCAI 2021 MSSEG-2 challenge (https://portal.fli-iam.irisa.fr/msseg-2/data/). Access was restricted to challenge participants. Requests to access these datasets should be directed to challenges-iam@inria.fr. The additional datasets used for validation and training of pipeline mediaire-B are not readily available because they were provided to mediaire GmbH by its customers' patients for the improvement and validation of its algorithms. mediaire GmbH does not hold the rights to distribute the data to third parties. Questions concerning these datasets should be directed to the corresponding author of this article.

## Ethics statement

Ethical review and approval was not required for the study on human participants in accordance with the local legislation and institutional requirements. The patients/participants provided their written informed consent to participate in this study.

## Author contributions

All authors listed have made a substantial, direct, and intellectual contribution to the work and approved it for publication.

## Funding

This project was funded partly by the European Regional Development Fund and the City Berlin (Grant number: 10631009926).

## Conflict of interest

All authors are employed at mediaire GmbH (as machine learning engineers or managers).

## Publisher's note

All claims expressed in this article are solely those of the authors and do not necessarily represent those of their affiliated organizations, or those of the publisher, the editors and the reviewers. Any product that may be evaluated in this article, or claim that may be made by its manufacturer, is not guaranteed or endorsed by the publisher.

## Footnotes

^1^ https://www.ofsep.org/en/hd-cohort

^2^ https://portal.fli-iam.irisa.fr/msseg-2/data/

^4^ https://files.inria.fr/empenn/msseg-2/Challenge_Day_MSSEG2_Introduction.pdf

^5^ https://adam.isi.uu.nl/

^6^ https://portal.fli-iam.irisa.fr/files/2021/06/MS_Challenge_Evaluation_Challengers.pdf

^7^ https://anima.irisa.fr/

^8^ https://zenodo.org/record/5775523#.YkVWKjxCRhE
